# Haemostatic and Biocompatibility Evaluation of Alginate-Functionalized Polylactide Composite Containing Zinc Sulphide and Hardystonite

**DOI:** 10.3390/md23090349

**Published:** 2025-08-29

**Authors:** Anna Kaczmarek, Zdzisława Mrozińska, Jerzy J. Chruściel, Michał Juszczak, Katarzyna Woźniak, Marcin H. Kudzin

**Affiliations:** 1 Łukasiewicz Research Network—Lodz Institute of Technology, 19/27 Marii Sklodowskiej-Curie Str., 90-570 Lodz, Poland; zdislawa.mrozinska@lit.lukasiewicz.gov.pl (Z.M.); marcin.kudzin@lit.lukasiewicz.gov.pl (M.H.K.); 2 Department of Molecular Genetics, Faculty of Biology and Environmental Protection, University of Lodz, 90-236 Lodz, Poland; michal.juszczak@biol.uni.lodz.pl (M.J.); katarzyna.wozniak@biol.uni.lodz.pl (K.W.)

**Keywords:** PLA, sodium alginate, hardystonite, zinc sulphide, blood plasma coagulation, genotoxicity, cell viability, DNA damage, PBM cells, Hs68 cells

## Abstract

The aim of this study was to evaluate the haemostatic potential and biocompatibility of a newly developed composite material for its use in blood-contacting applications. Based on promising reports on polylactide (PLA), sodium alginate (ALG), and bioactive additives such as hardystonite (HT) and zinc sulphide (ZnS), a melt-blown PLA nonwoven was modified via dip-coating using an ALG solution as a matrix for incorporating HT and ZnS particles, resulting in the PLA-ALG-ZnS-HT composite. The material was characterised in terms of surface morphology, specific surface area, pore volume, average pore size, and zeta potential (pH~7.4). Haemostatic activity was assessed by measuring blood coagulation parameters, while biocompatibility was evaluated through the viability of human peripheral blood mononuclear (PBM) cells and human foreskin fibroblasts (Hs68). Genotoxicity was analysed using the comet assay and plasmid relaxation test. Results confirmed a uniform alginate coating with dispersed HT and ZnS particles on PLA fibres. The modification increased the surface area and pore volume and caused a shift toward less negative zeta potential. Haemostatic testing showed prolonged activated partial thromboplastin time (aPTT), likely due to Zn^2+^ interactions with clotting factors. Biocompatibility tests showed high cell viability and no genotoxic effects. Our findings suggest that the PLA-ALG-ZnS-HT composite is safe for blood and skin cells and may serve as an anticoagulant material.

## 1. Introduction

Polymers constitute a diverse and rapidly advancing class of materials with wide-ranging applicability, particularly in the biomedical and healthcare sectors. Their use is prominent in tissue engineering, wound care, and biotechnological applications [1,2,3,4].

Among synthetic polymers, polyglycolic acid is a notable example, utilised as a scaffold for cartilage and blood vessel regeneration [3], as well as in the treatment of open soft tissue wounds [2]. It has also been approved by the FDA (Food and Drug Administration) for use in medical applications, including biodegradable sutures and other implantable devices, similar to polylactide [2].

Polylactide, in turn, is incorporated into a variety of implantable medical components, such as screws, pins, rods, orthopaedic supports, and surgical meshes [3]. At present, numerous polylactide-based orthopaedic products are available on the market, including the Full Thread Bio Interference Screw^®^ (Arthrex), Meniscal Stinger^®^ (Linvatec), BioScrew^®^ (Conmed), Phantom Soft Thread Soft Tissue Fixation Screw^®^ (DePuy), Bio-Anchor^®^ (Conmed), Phantom Suture Anchor^®^ (DePuy), and Clearfix Meniscal Dart^®^ (Innovasive Devices) [2]. Furthermore, this polymer is employed in drug delivery platforms and in the regeneration of bone and other tissues [3].

Another commonly used synthetic material is polycaprolactone, primarily serving as a scaffold for bone and cartilage repair [3]. It is also suitable for long-term implants, dental splints, and as a carrier in controlled drug release systems [2,3].

Among natural polymers, collagen has found widespread use in skin regeneration, tissue engineering matrices, haemostatic agents, and treatment of burns and wounds, as well as in pharmaceutical delivery [2,3]. Chitosan is likewise frequently applied in biomedical scaffolds and drug transport systems, particularly in the form of hydrogels and microspheres [2,3]. Gelatin also plays a significant role in tissue engineering and drug administration [3]; additionally, it is used as a capsule shell material [3] and in various types of wound dressings [2].

In response to the growing demand for innovative materials, hybrid polymer systems have emerged as a promising solution, with composite structures receiving particular attention due to their controllable and enhanced functional characteristics [1,2,3,4].

Among synthetic polymers used in biomedical applications, poly (lactic acid) (PLA) has garnered considerable interest [5,6,7,8,9]. Owing to its biodegradability, biocompatibility, and mechanical versatility, PLA is frequently employed in drug delivery platforms, wound dressings, and regenerative medicine [9,10,11,12,13,14,15,16,17,18]. Moreover, PLA primarily undergoes degradation via hydrolysis, generating lactic acid as a by-product, which is naturally metabolised and cleared from the body without adverse effects [5,8,9]. In addition to its environmental sustainability, PLA offers favourable thermoplastic processability, suitable mechanical strength [8,19], and a non-toxic profile [19,20,21]. However, limitations such as brittleness, hydrophobic surface characteristics, and the absence of functional side groups may restrict its broader biomedical utility [22]. These limitations have prompted the development of PLA-based composites incorporating functional biomolecules and biopolymers.

One such biopolymer is alginate (ALG), a naturally occurring anionic polysaccharide composed of alternating sequences of β-D-mannuronic acid (M) and α-L-guluronic acid (G) residues [23,24]. Typically sourced from brown seaweed, alginate can also be biosynthesised by certain bacterial strains [24,25,26,27,28,29,30,31,32,33]. Its hydrophilic nature and water solubility, combined with its mild gelation properties, make alginate especially suitable for biomedical formulations [24,25,26,27,28,29,30,31,32,33].

Alginate’s widespread use is attributed to its biocompatibility, biodegradability, non-toxicity, chemical versatility, non-immunogenicity, and intrinsic bioactivity [24,26,27,28,29,30,31,32,33,34]. Being the second-most abundant biopolymer after cellulose, it is relatively inexpensive to extract and process [26]. The presence of numerous hydroxyl and carboxyl groups in its molecular structure facilitates chemical modification and ionic crosslinking [27,29,32], while its ability to undergo sol–gel transitions makes it particularly attractive for hydrogel and coating applications [24,27]. Alginate has thus been widely employed in textile engineering and surface functionalisation through methods such as layer-by-layer deposition, ionic crosslinking, and nanocomposite coating techniques [27].

Given these advantageous physicochemical and biological properties, alginate has gained prominence in wound healing and tissue engineering research [23,25,33,34,35,36]. Alginate-based dressings are capable of absorbing wound exudate efficiently while inhibiting microbial growth [28]. Due to their high water content, they maintain a moist environment and stable pH levels conducive to accelerated tissue regeneration [24,33,37]. Moist wound conditions have been demonstrated to significantly enhance healing outcomes [38]. Furthermore, alginate exhibits a high fluid absorption capacity—reported to be 2.2 times its weight [30]—and forms hydrogels in situ, contributing to its unique haemostatic properties [30]. Studies also suggest its capacity to stimulate epidermal regeneration [24], further highlighting its therapeutic potential in wound care and haemostasis [30,33,36,37].

To augment the bioactivity of PLA-based systems, bio-ceramic additives such as hardystonite (HT)—a calcium-zinc silicate—have been introduced. In a study conducted by Zhang et al. [39], a Janus-type bilayer membrane was engineered by incorporating hydrophilic HT particles into hydrophobic PLA fibrous layers. Fabricated via hot pressing, the resulting membrane facilitated directional fluid transport, with the PLA layer acting as a protective barrier, thereby preventing direct exposure of the wound to the ceramic phase. In vivo evaluation using a rat burn model demonstrated superior healing outcomes, including enhanced proliferation of dermal papilla cells, improved epithelialisation, and increased cell viability and migration. These effects were largely attributed to the synergistic release of Zn^2+^ and SiO_3_^2−^ ions, and their combined action was more efficacious than either ion alone.

Another investigation [40] explored the incorporation of HT into an electrospun membrane composed of polycaprolactone (PCL) and tetrafluoroethylene (TPE). HT not only improved cellular activity—enhancing migration, proliferation, and differentiation—but also influenced fibre morphology. Specifically, the presence of Ca^2+^ and Zn^2+^ increased the electrical conductivity of the spinning solution, resulting in greater electrostatic stretching and reduced fibre diameter. However, higher HT content simultaneously elevated the solution viscosity, leading to thicker fibres. These results underscore the importance of balancing ionic conductivity and rheological behaviour in electrospun scaffold fabrication. Additionally, membranes produced via near-field electrospinning exhibited superior oxygen permeability compared to cast films, thereby further enhancing the wound healing potential.

A related study [41] investigated an injectable hydrogel system composed of sodium alginate and hardystonite. In vitro tests using human umbilical vein endothelial cells (HUVECs) and human dermal fibroblasts (HDFs) confirmed the biocompatibility and functionality of the hydrogel network. The incorporation of HT at 2 wt.% enhanced compressive strength and accelerated gelation. Importantly, the composite hydrogels significantly enhanced cellular proliferation, migration, and viability compared to pure alginate controls. Additionally, the release of Zn^2+^ ions conferred robust antibacterial activity, achieving full inhibition of microbial growth in both direct-contact and extract-based assays.

Beyond hardystonite, recent research has increasingly focused on zinc-based degradable biomaterials due to their bioactivity, degradability, and regenerative potential [42]. Zinc sulphide (ZnS) nanoparticles have emerged as promising agents in tissue engineering and wound therapy [43,44,45,46,47,48,49,50]. ZnS functions as a hydrogen sulphide (H_2_S) donor, undergoing degradation under acidic conditions to release both Zn^2+^ and H_2_S [51,52]. Zn^2+^ offers well-established antimicrobial properties, while H_2_S enhances the inflammatory response, promotes neovascularisation, and expedites tissue repair processes [51,52].

ZnS nanoparticles have also demonstrated the ability to stimulate stem cell proliferation and differentiation—key processes in tissue regeneration [46]. Furthermore, they promote the activity of critical cell types such as fibroblasts and keratinocytes, which are essential for wound closure [49]. In addition to their regenerative capabilities, ZnS nanoparticles exhibit potent antimicrobial efficacy against a broad spectrum of pathogens, including *Staphylococcus aureus*, *Bacillus subtilis*, *Escherichia coli*, and *Pseudomonas aeruginosa*, as well as fungal strains such as *Candida albicans* and *Aspergillus niger* [53].

Based on the promising data reported in the literature regarding the individual properties of polylactide, sodium alginate, and bioactive additives such as hardystonite and zinc sulphide, the authors decided to combine these components to develop a novel composite material for wound dressing applications. The melt-blown PLA nonwoven was modified with an ALG solution serving as a matrix for the incorporation of HT and ZnS, resulting in the composite (PLA-ALG-ZnS-HT). The main objective of this study was to evaluate the haemostatic potential and biocompatibility of the developed material for its use as an advanced bioactive material intended for wound treatment and/or other blood-contacting applications. To this end, the developed composite was assessed in terms of physicochemical characteristics, as well as the haemostatic potential. Biocompatibility was analysed by investigating the effects of post-incubation PLA composites mixtures on the cell viability and DNA (deoxyribonucleic acid) damage in peripheral blood mononuclear (PBM) cells and human foreskin fibroblasts cells (Hs68 cell line).

## 2. Results and Discussion

### 2.1. Zinc and Calcium Concentration

The concentrations of zinc and calcium in the developed PLA-ALG-ZnS-HT composite were assessed using inductively coupled plasma mass spectrometry (ICP-MS). It is of the utmost importance to assess the content of these two elements, since they may play a crucial role in the composite’s bioactivity. The measured calcium (Ca) content in the composite after the dip-coating procedure was 10,392 ± 1025 mg/kg, while the zinc (Zn) content was 192,512 ± 1131 mg/kg. It can be observed that the Zn content is substantially higher than that of Ca. This may be easily explained by the fact that zinc was present in both dip-coating suspensions, whereas calcium was present only in the hardystonite suspension. Therefore, the resulting concentration of zinc is much higher than that of calcium.

### 2.2. Surface Morphology

**Figure 1:**
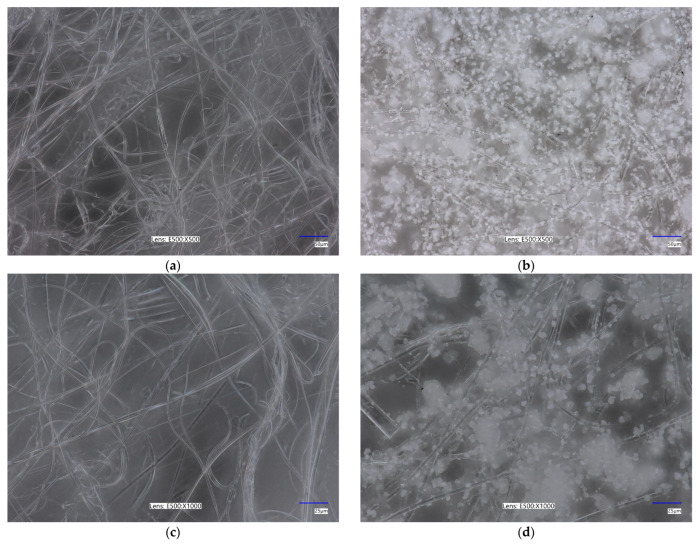
Optical microscopy images of unmodified (**a**,**c**) and modified PLA nonwovens (**b**,**d**). Magnification 500× (**a**,**b**) and 1000× (**c**,**d**).

The surface morphology of the unmodified PLA nonwoven and the PLA-ALG-ZnS-HT composite was examined using an optical microscope and is presented in Figure 1. In the case of the unmodified PLA nonwoven (Figure 1a,c), a typical three-dimensional fibrous structure was observed. The observed micro- and nanofibers form a porous structure with interconnected pores, which is characteristic for the melt-blown nonwovens. The analysed melt-blown fibres exhibit random orientation and varying diameters.

A similar structure can be observed for the PLA-ALG-ZnS-HT composite (Figure 1b,d). In the modified sample, numerous agglomerates of ZnS and HT were noticed around the PLA fibres. These findings confirm that ZnS and HT particles were successfully incorporated into the sodium alginate matrix, forming a coating that encapsulated the PLA fibres. The abundance of visible agglomerates is consistent with the elevated concentrations of Ca and Zn in the composite.

### 2.3. Specific Surface Area, Total Pore Volume, and Average Pore Size

**Figure 2:**
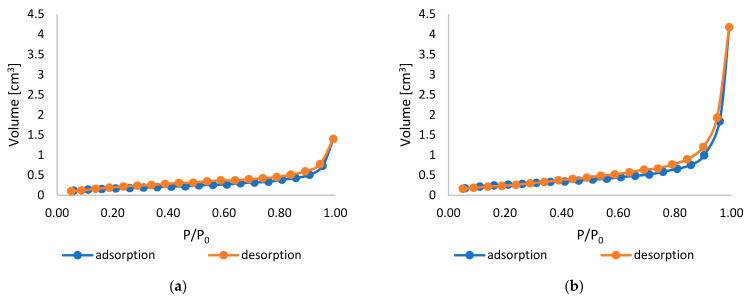
Adsorption and desorption isotherms of unmodified (**a**) and modified PLA nonwovens (**b**).

The specific surface area (SSA), total pore volume (TPV), and average pore size (APS) were determined using the Brunauer–Emmett–Teller (BET) method. The calculated SSA, TPV, and APS values are presented in Table 1, while Figure 2 shows the adsorption–desorption isotherms recorded for unmodified and modified PLA nonwovens.

According to the data presented in Table 1, the specific surface area increased from 1.082 m^2^/g for the unmodified PLA nonwoven to 1.217 m^2^/g for the PLA-ALG-ZnS-HT composite. This may be due to the presence of abundant ZnS and HT particles on the surface of the modified sample. The presence of multiple nano- and micro-sized agglomerates results in a more developed and rougher surface, which, in turn, leads to a larger specific surface area. Similarly, the total pore volume of the PLA nonwoven increased from 3.985 × 10^−3^ cm^3^/g to 8.210 × 10^−3^ cm^3^/g after the dip-coating procedure. This may be due to the structural changes caused by the dip-coating procedure, which could lead to the expansion of the fibre network. During the dip-coating process, air entrapment may occur, creating additional voids between the fibres and enhancing the inter-fibre spacing. This could likewise account for the increase in the average pore size (from 14.73 nm for the unmodified PLA to 26.99 nm in the case of the PLA-ALG-ZnS-HT), which further contributes to the enhanced total pore volume. Additionally, during the drying process, as the solvent evaporates, some cracks and voids may also appear, contributing to the rise in the overall porosity of the sample. Similar results were previously obtained by Kudzin et al. [54], who modified PLA melt-blown nonwovens via the dip-coating method using sodium alginate and ZnCl_2_ solution. The authors observed the increased specific surface area and total pore volume after the coating procedure due to the higher mesoporosity.

As shown in Figure 2, the obtained curves exhibit a characteristic S-shaped profile, which, in accordance with IUPAC (International Union of Pure and Applied Chemistry) classification, corresponds to type II isotherms. This type of isotherm occurs in the case of monolayer–multilayer adsorption [55,56]. For both the unmodified and modified PLA samples, the onset of the nearly linear middle segment, i.e., Point B, is not very distinctive. This suggests the overlap of the monolayer formation with the multilayer adsorption [55,56]. The general shape of the curves indicates that mesoporous and macroporous structures dominate, while micropores are less pronounced in the materials [57]. The exponential increase in the quantity of adsorbed nitrogen with the rising relative pressure further supports this interpretation; initially (at low pressures), nitrogen penetrates the micropores, followed by monolayer formation and subsequent multilayer accumulation at higher pressures [57].

A hysteresis loop was present in the isotherms of all analysed samples, which is indicative of mesoporous structures and capillary condensation occurring within mesopores [55,56]. The observed loops are consistent with type H3, commonly associated with slit-shaped pores [57]. Moreover, such hysteresis is often associated with the aggregates of plate-like particles or when pore filling is incomplete in larger pores, particularly macropores [56]. In the case of the PLA-ALG-ZnS-HT, the hysteresis loop is noticeably larger, indicating higher mesoporosity.

### 2.4. Zeta Potential

The zeta potential of the examined PLA and PLA-ALG-ZnS-HT samples was measured at the physiological pH (~7.4). For the unmodified PLA nonwoven, a highly negative zeta potential value was measured, equal to −77.96 ± 0.41 mV. The modification with the ZnS and HT resulted in the shift towards a less negative value, i.e., −50.69 ± 0.37 mV. The negative zeta potential of the PLA nonwovens results from the presence of free carboxylic acid groups at the end of the PLA polymer chain [58]. The less negative zeta potential of the PLA-ALG-ZnS-HT arises from the addition of hardystonite and zinc sulphide. Both of those substances contain positively charged ions, such as Ca^2+^ and Zn^2+^, which may accumulate near the surface, neutralising the negative zeta potential to some extent. Likewise, sodium alginate includes the Na^+^ ions, which are likely to have a corresponding impact.

The analysis of the zeta potential at the physiological pH is crucial, as it plays a significant role in the material’s interaction with proteins and blood components, thereby influencing blood plasma coagulation, as well as biological activity.

### 2.5. Blood Plasma Coagulation: Activated Partial Thromboplastin Time, Prothrombin Time, and Thrombin Time

**Figure 3:**
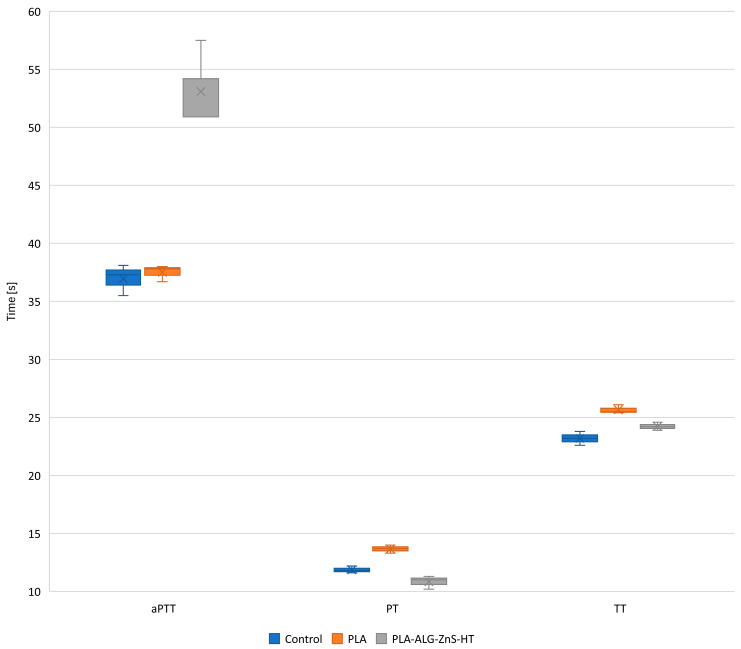
The aPTT, PT, and TT measured for the unmodified and modified PCL nonwovens after 1 h of incubation at 37 °C.

Assessing the hemocompatibility of the developed PLA composite is essential for its potential use in biomedical applications, such as wound dressings. To evaluate its effects on blood clotting and thrombogenic potential, in vitro coagulation tests were conducted. These tests included measurements of activated partial thromboplastin time (aPTT), prothrombin time (PT), and thrombin time (TT). The aPTT test is a common laboratory procedure used to examine the integrity of the intrinsic coagulation pathway [59,60,61]. It determines the duration required for plasma to clot following the addition of an activator (e.g., kaolin or silica), phospholipids, and calcium ions [59,60,61]. The intrinsic pathway encompasses coagulation factors XII, XI, IX, and VIII [59,60,61]. Conversely, PT evaluates the extrinsic coagulation pathway by measuring clotting time after adding a tissue factor (thromboplastin) to plasma [59,60,61], with factor VII playing a key role in this pathway [59,60,61]. Finally, TT is used to assess the conversion of fibrinogen to fibrin. It is influenced only by thrombin- and fibrinogen-related factors. By analysing all these parameters, the influence of PLA and PLA-ALG-ZnS-HT on different coagulation cascades can be comprehensively assessed, providing insights into their hemocompatibility and thrombogenic risk. The results of these assays are shown in Figure 3.

PLA is generally considered biocompatible and relatively inert. Therefore, it is not expected to observe any direct effect on the blood plasma coagulation parameters. This is consistent with the obtained results, which showed that PLA did not affect the aPTT, while the impact on PT and TT was minimal. The observed minimal effect of unmodified PLA on the PT may be due to interactions between the negatively charged surface with coagulation factors, which may lead to their inactivation. In the case of the TT, the highly negative zeta potential can cause its prolongation due to interference with thrombin–fibrinogen interactions, as well as due to the adsorption of thrombin.

On the other hand, the PLA-ALG-ZnS-HT composite resulted in the prolonged aPTT and slightly shortened PT and TT. In general, calcium ions are regarded as essential cofactors in blood coagulation [62,63,64], and hence, the presence of calcium in the hardystonite theoretically should result in accelerated clotting via the intrinsic pathway, leading to a shortened aPTT. Nevertheless, in the case of the PLA-ALG-ZnS-HT composite, the opposite effect was observed. This may be because the content of zinc in the composite is much higher than the content of calcium. Therefore, the effect associated with the Zn^2+^ ions is dominant. According to the literature, the transition metals, such as copper, cobalt, nickel and zinc, may bind to the XI, XII, and HK contact factors present in human plasma, resulting in the prolongation of the aPTT [65]. This phenomenon might account for the observed change in the aPTT in the case of the PLA-ALG-ZnS-HT composite. Similar results were obtained in our previous works [54,66]. At the same time, the observed effect on the PT and TT is negligible, since all the measured PT and TT times were within the reference range. This leads to the conclusion that only the intrinsic coagulation pathway was affected by the PLA-ALG-ZnS-HT composite.

### 2.6. Viability of PBM and Hs68 Cells

**Figure 4:**
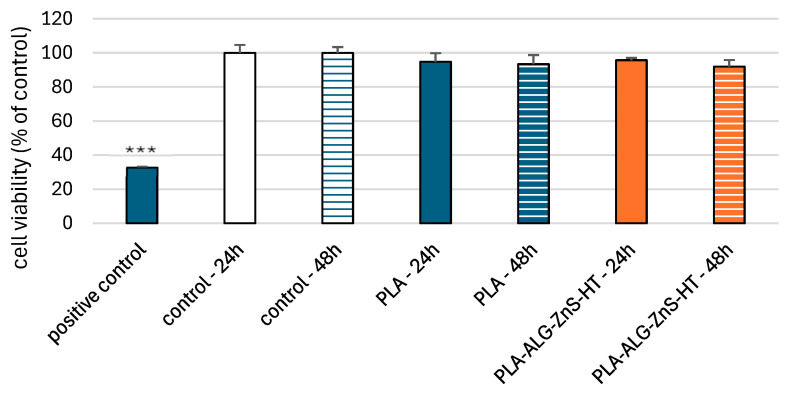
PBM cells viability results after 24 and 48 h of incubation for unmodified PLA and PLA-ALG-ZnS-HT post-incubation mixtures. Presented as the mean result after 6 repeats. Error bars denote SD (standard deviation); *** *p* < 0.001 vs. control—24 h.

The resazurin reduction assay was used to determine cell viability after incubation with post-incubation mixtures of unmodified PLA and PLA-ALG-ZnS-HT. This assay is based on the application of an indicator dye to measure oxidation-reduction reactions, which principally occur in the mitochondria of live cells. The non-fluorescent dark blue dye (resazurin) is reduced by metabolically active cells to resorufin, a fluorescent compound that appears pink at 570 nm and red at neutral pH. We showed that incubation of PBM cells with PLA-ALG-ZnS-HT post-incubation mixtures did not decrease cell viability after 24 and 48 h (Figure 4).

**Figure 5:**
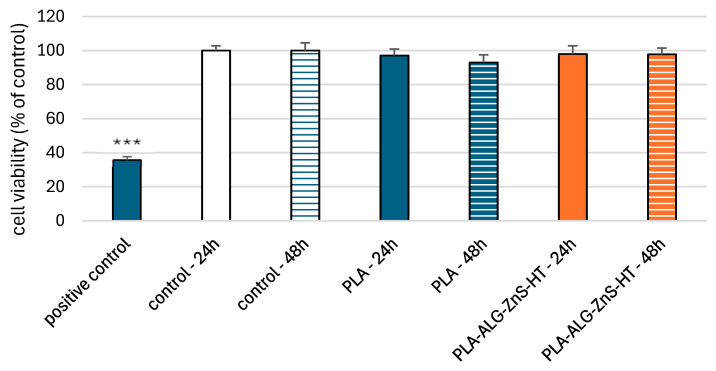
Hs68 cells viability after 24 and 48 h of incubation for unmodified PLA and PLA-ALG-ZnS-HT post-incubation mixtures. Presented as the mean result after 6 repeats. Error bars denote SD; *** *p* < 0.001 vs. control—24 h.

We obtained comparable results for Hs68 cells (Figure 5). Our results indicate the absence of cytotoxic properties of the PLA-ALG-ZnS-HT composite against both types of cells. These results suggest that hardystonite and zinc sulphide present in the tested composite do not cause cytotoxic effects in normal cells. Hardystonite stimulates cells of the MG-63 cell line, which may indicate the compound’s potential to promote bone regeneration [67]. Similar results were obtained for the MC3T3-E1 cell line [68].

### 2.7. DNA Damage in PBM Cells and Hs68 Cells

**Figure 6:**
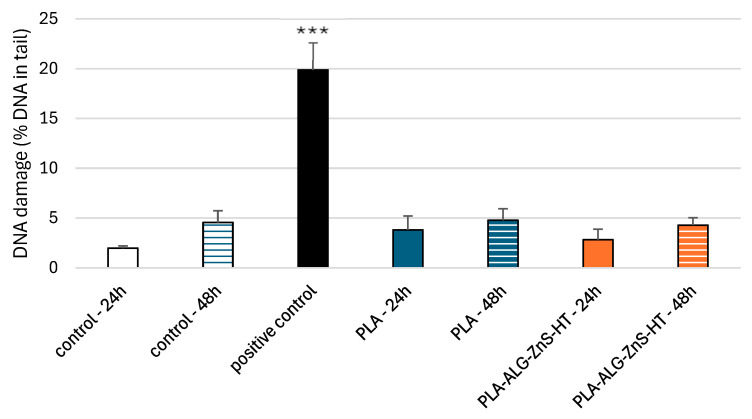
DNA damage in PBM cells after 24 and 48 h of incubation for unmodified PLA and PLA-ALG-ZnS-HT post-incubation mixtures. Presented as the mean result after 100 comets. Error bars denote SEM (standard error mean); *** *p* < 0.001 vs. control—24 h.

**Figure 7:**
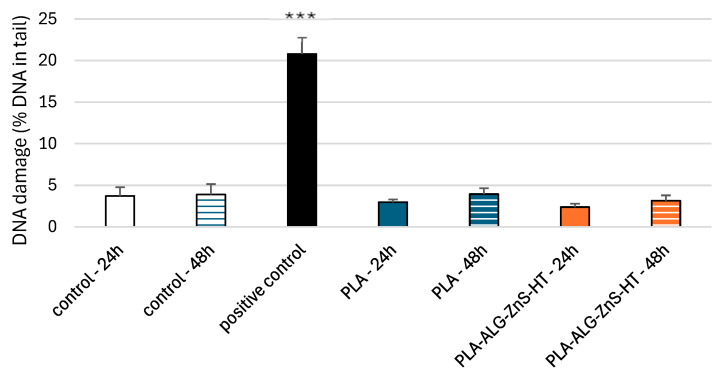
DNA damage in Hs68 cells after 24 and 48 h of incubation for unmodified PLA and PLA-ALG-ZnS-HT post-incubation mixtures. Presented as the mean result after 100 comets. Error bars denote SEM; *** *p* < 0.001 vs. control—24 h.

The comet assay in the alkaline version is a sensitive and simple method for determining the level of DNA damage, including single- and double-stranded breaks and alkali-labile sites in living cells [69]. We observed severe DNA damage in PBM cells and Hs68 cells incubated with 25 µM H_2_O_2_ (hydrogen peroxide), i.e., the positive control. In the case of PLA-ALG-ZnS-HT post-incubation mixtures, no increase in DNA damage was observed after 24 and 48 h of incubation in either cell line (Figure 6 and Figure 7).

**Figure 8:**
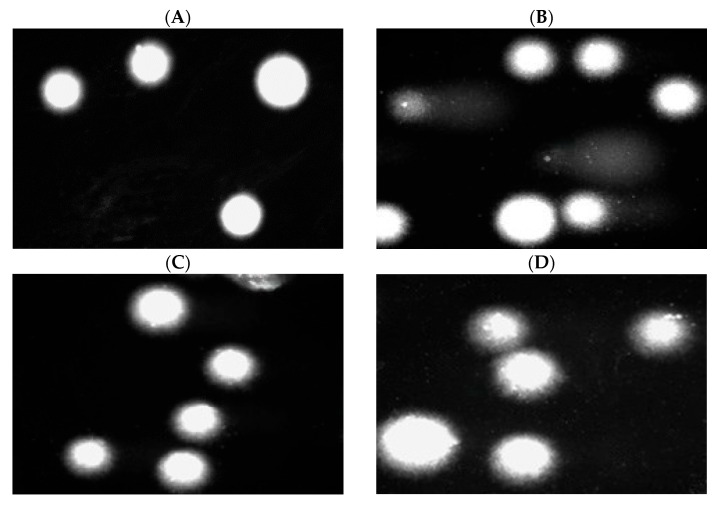
Effect of medium (**A**), 25 µM hydrogen peroxide (**B**), PLA (**C**), and PLA-ALG-ZnS-HT (**D**) post-incubation mixtures on DNA damage in PBM cells after 24 h of incubation.

**Figure 9:**
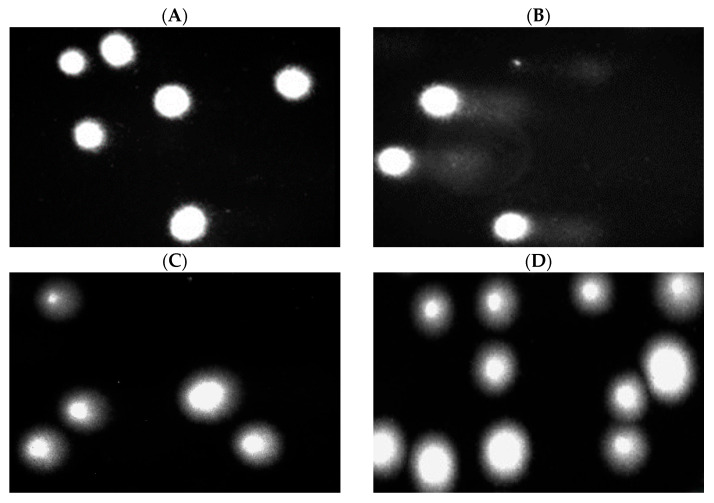
Effect of medium (**A**), 25 µM hydrogen peroxide (**B**), PLA (**C**), and PLA-ALG-ZnS-HT (**D**) post-incubation mixtures on DNA damage in Hs68 cells after 24 h of incubation.

Moreover, we present pictures of comets (Figure 8 and Figure 9) that show no DNA damage in the case of post-incubation mixtures, whereas DNA damage induced by hydrogen peroxide is clearly visible. Our results show that the tested PLA-ALG-ZnS-HT composite did not exhibit DNA damage. Although zinc sulphide can induce DNA damage in leukemic cells, no such effect was observed in normal cells [70]. Additionally, a study conducted on mE-ASCs cells, which can differentiate into bone cells, indicates the absence of cytotoxic and genotoxic effects of hardystonite [71].

### 2.8. Plasmid Relaxation Assay

**Figure 10:**
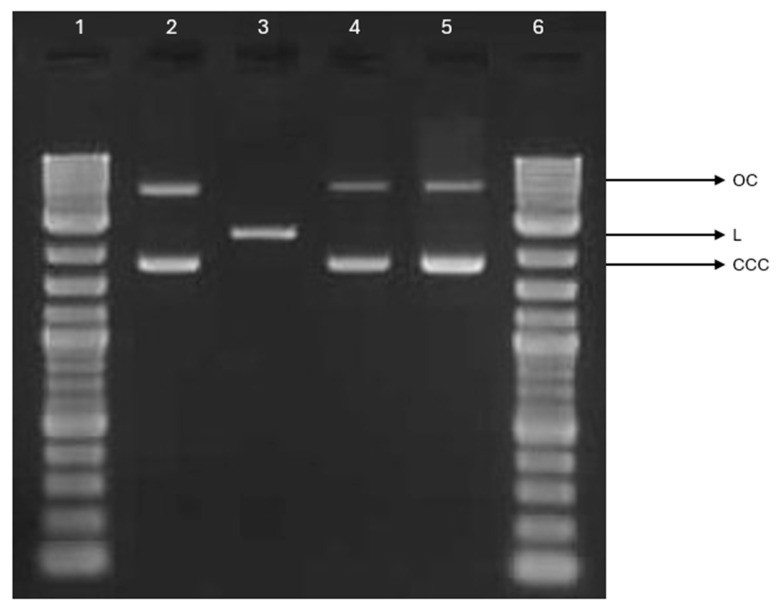
Plasmid relaxation assay. pUC19 plasmid was incubated for 24 h (37 °C) with alginate-functionalised PLA nonwoven post-incubation mixtures (PLA and PLA-ALG-ZnS-HT), then was separated on a 2% agarose gel, stained with ethidium bromide, and visualised in UV (ultraviolet) light. Line 1—DNA ladder; line 2—pUC19 plasmid (the supercoiled form); line 3—pUC19 plasmid incubated with restrictase *Pst*I (the linear form); lines 4 and 5—pUC19 plasmid incubated with PLA and PLA-ALG-ZnS-HT, respectively; line 6—DNA ladder. OC—the open circular form of plasmid DNA.

The potential interactions between PLA-ALG-ZnS-HT post-incubation mixtures and DNA were investigated using the plasmid relaxation assay. Electrophoretic mobility shift analysis (EMSA) revealed that the pUC19 plasmid, isolated from DH5α *Escherichia coli*, predominantly exhibited a supercoiled conformation (CCC), characterised by high electrophoretic mobility. To obtain a form with altered electrophoretic mobility, the plasmid was linearised by overnight incubation with the restriction enzyme *Pst*Iat 37 °C, yielding the linear (L) form (Figure 10). Neither unmodified PLA nor PLA-ALG-ZnS-HT interacted with plasmid DNA, and no single- or double-stranded breaks were observed. Our results demonstrate that that the post-incubation mixture does not interact with DNA.

## 3. Materials and Methods

### 3.1. Materials

Poly (lactic acid), type Ingeo™ 3251D, with a melt flow rate (MFR) of 30–40 g/10 min (190 °C, 2.16 kg), was provided by NatureWorks LLC (Minnetonka, MN, USA).

Alginic acid sodium salt (C_5_H_7_O_4_COONa; CAS: 9005-38-3) was purchased from Merck KGaA (Darmstadt, Germany).

Hardystonite (Ca_2_ZnSi_2_O_7_), in a form of powder (0–80 μm), was purchased from Matexcel (Shirley, NY, USA).

Zinc sulphide (CAS: 1314-98-3), in a form of powder (10 μm), was purchased from Merck KGaA (Darmstadt, Germany).

The human blood plasma lyophilizates (Dia-CONT I), as well as the reagents Dia-PTT, Dia-PT, Dia-TT, and calcium chloride solution (0.025 M CaCl_2_), were obtained from Diagon Kft (Budapest, Hungary).

The human foreskin fibroblast line Hs68 (ATCC^®^ CRL-1635™) was obtained from the American Type Culture Collection (ATCC™, Manassas, VA, USA).

Hydrogen peroxide (MQ100; CAS: 7722-84-1) was purchased from Merck KGaA (Darmstadt, Germany).

Doxorubicin (MQ200; CAS: D1515) was purchased from Merck KgaA (Darmstadt, Germany).

### 3.2. Methods

#### 3.2.1. Sample Preparation via Melt-Blowing Technique and Dip-Coating Method

Poly (lactic acid) nonwoven samples were fabricated using the melt-blowing method with a laboratory-scale single-screw extruder (Axon, Limmared, Sweden). The extruder was equipped with a die head containing 30 holes, each with a diameter of 0.25 mm. The specific processing conditions used for the preparation of the PLA nonwovens are detailed in Table 2.

The PLA melt-blown nonwoven was modified by a three-step dip-coating process. Firstly, the PLA sample was immersed in a 0.5% sodium alginate solution for one minute to ensure a thorough coating. The 0.5% sodium alginate solution was prepared by dissolving 0.5 g of alginic acid sodium salt (Merck KGaA, Darmstadt, Germany) in 100 mL of deionised water. The mixture was stirred magnetically at 45 °C until a homogeneous solution was obtained. Afterwards, the sample was immediately transferred to a 10% ZnS suspension and immersed for one minute. The 10% ZnS suspension was prepared by adding 10 g of ZnS (Merck KGaA, Darmstadt, Germany) to 100 mL of deionised water and stirring magnetically at room temperature. Finally, the sample was immersed for one minute in 10% Ca_2_ZnSi_2_O_7_ suspension, prepared by adding 10 g of Ca_2_ZnSi_2_O_7_ (Matexcel, Shirley, NY, USA) to 100 mL of deionised water and stirred magnetically at room temperature. After the dip-coating procedure, the samples were gently pressed and dried at 50 °C until a constant weight was reached. As a result, two types of samples were obtained: unmodified PLA nonwoven (denoted as PLA) and modified PLA composite (denoted as PLA-ALG-ZnS-HT).

#### 3.2.2. Zinc and Calcium Concentration

To assess the zinc and calcium contents, the composite samples were mineralised using a Magnum II microwave mineraliser (Ertec, Wrocław, Poland) equipped with a single module. Sample digestion was conducted in a sealed system, allowing precise control of both temperature and pressure. The process involved adding 2.5 mL of 65% nitric acid and 2.5 mL of hydrogen peroxide. The concentration of each analysed element was then quantitatively determined using an ICP-MS 7900 instrument (Agilent Technologies, Santa Clara, CA, USA). Each measurement was performed twice, and the final reported values represent the average of the two measurements.

#### 3.2.3. Surface Morphology

The surface morphology of the samples was examined using an optical microscope. Optical microscopy was conducted with a VHX-7000N digital microscope (Keyence, Osaka, Japan) at magnifications of 500× and 1000×. To acquire images, high dynamic range imaging and Z-stacking were applied.

#### 3.2.4. Specific Surface Area, Total Pore Volume, and Average Pore Size

The specific surface area, total pore volume, and average pore size were determined using the Brunauer–Emmett–Teller method. The measurements were performed with an Autosorb-1 analyser (Quantachrome Instruments, Boynton Beach, FL, USA), employing nitrogen at 77 K as the adsorbate. Prior to testing, the samples were degassed at room temperature for 24–48 h. Each measurement was conducted using approximately 0.5–0.8 g of the sample.

#### 3.2.5. Zeta Potential

Zeta potentials of the samples were determined using the commercially available streaming potential analyser SurPASS 3 (Anton Paar, Graz, Austria). Experiments were conducted at room temperature, with a phosphate-buffered saline (PBS, pH~7.4) used as an electrolyte. The applied pressure range was 200–600 mbar, and the upper pressure limit used for calculation of the zeta potential was set to 400 mbar. Measurements were made using a cylindrical cell. Five measurements were taken for each sample.

#### 3.2.6. Blood Plasma Coagulation: Activated Partial Thromboplastin Time, Prothrombin Time, and Thrombin Time

The impact of the developed composites on blood plasma coagulation was analysed by determining the activated partial thromboplastin time, prothrombin time, and thrombin time. These parameters were measured using the Coag4D coagulometer (Diagon Kft, Budapest, Hungary). Samples (~10 mg) were incubated at 37 °C for 1 h in 250 µL of plasma derived from commercially available lyophilised human plasma (Dia-CONT I, Diagon Kft, Budapest, Hungary). The aPTT tests were conducted using the Dia-PTT reagent and 0.025 M calcium chloride solution (Dia-CaCl_2_), both provided by Diagon Kft (Budapest, Hungary), according to the manufacturer’s protocol. For PT testing, the Dia-PT reagent (Diagon Kft, Budapest, Hungary) was used, while TT tests were performed using the Dia-TT reagent (Diagon Kft, Budapest, Hungary). All experiments were performed in triplicate. The full procedure has been described in detail in our previous publications [66,72,73,74].

#### 3.2.7. Preparation of Samples for the Assessment of Biological Properties

To analyse the influence of PLA-ALG-ZnS-HT nonwoven composites incorporating hardystonite and zinc sulphide on cells, 1 cm^2^ samples of modified and unmodified PLA nonwovens were incubated with 3 mL of RPMI (Roswell Park Memorial Institute) or DMEM (Dulbecco’s Modified Eagle Medium) medium, depending on the cell line, at 37 °C in 5% CO_2_ for 24 h. After incubation, the post-incubation mixtures were filtered using a 0.2 µm filter to ensure aseptic conditions. For the plasmid relaxation assay, 1 cm^2^ samples of modified and unmodified PLA nonwovens were incubated in molecular-grade water. The resulting polylactide post-incubation mixtures were then added to cells at a 1:9 ratio to assess their influence on cell viability, DNA damage, and plasmid relaxation.

#### 3.2.8. Cell Culture

Peripheral blood mononuclear cells were isolated from a leucocyte-rich buffy coat collected from the blood of healthy non-smoking donors from the Blood Bank inLodz, Poland, as described previously [75]. The first step of PBM cells isolation involved mixing fresh buffy coat blood with PBS at a 1:1 ratio. Next, the mixture was centrifuged over a density gradient of Lymphosep (Cytogen, Zgierz, Poland) at 2200 rpm for 20 min using the lowest acceleration and deceleration settings. The PBM cells were then washed three times by centrifugation with 1% PBS. After isolation, cells were suspended in RPMI 1640 medium. This study was approved by the University of Lodz Research Ethics Committee (12/KEBN-UŁ/I/2024-2025).

The human foreskin fibroblast line Hs68 (ATCC^®^ CRL-1635™) was obtained from the American Type Culture Collection (ATCC™, Manassas, VA, USA). Hs68 cells were cultured in high-glucose DMEM supplemented with 100 units/mL potassium penicillin, 100 μg/mL streptomycin sulphate, and 10% (*v*/*v*) foetal bovine serum (FBS). Cultures were maintained at 37 °C in a humidified atmosphere with 5% CO_2_.

#### 3.2.9. Cell Viability Resazurin Assay

The cell viability resazurin assay was performed using the method described by O’Brien et al. [76]. Resazurin salt powder was dissolved in a sterile PBS buffer. Post-incubation mixtures were added to PBM cells at a density of 5 × 10^4^ cells and to Hs68 cells at a density of 1 × 10^4^ cells, then incubated for 24 and 48 h at 37 °C in 5% CO_2_. The control was RPMI 1640 for PBM cells and DMEM for Hs68 cells, prepared in the same manner as the post-incubation mixtures. As a positive control, PBM and Hs68 cells incubated for 24 h with 50 μM doxorubicin were used. This allowed for the evaluation of the cytotoxic effects of the test materials in comparison to a well-established cytotoxic agent. Next, 10 µL of resazurin salt solution was added to each well, and the plates were incubated again at 37 °C in 5% CO_2_ for 2 h. Fluorescence was then measured with the HT microplate reader BioTek Synergy HT (Agilent Technologies, Inc., Santa Clara, CA, USA) using excitation/emission wavelengths of λ_ex_  =  530/25 and λ_em_  =  590/35 nm. The effects of the polylactide post-incubation mixtures were quantified as the percentage of control fluorescence.

Cell viability after incubation with the test materials is presented as the mean ± SD. Statistical analysis was performed using a two-tailed Student’s *t*-test. Statistical significance levels refer to comparisons between the experimental samples (PLA and PLA-ALG-ZnS-HT) and the corresponding controls after the appropriate incubation time. Additionally, the positive controls were compared to their respective controls after 24 h of incubation. Each cell type had its own dedicated control group. Differences were considered statistically significant at *p* < 0.05.

#### 3.2.10. DNA Damage

PLA and PLA-ALG-ZnS-HT post-incubation mixtures were added to PBM cells at the density of 7.5 × 10^4^ cells and to Hs68 cells at the density of 3.75 × 10^4^ cells, then incubated for 24 and 48 h at 37 °C in 5% CO_2_. The negative control was RPMI 1640 for PBM cells and DMEM for Hs68 cells, prepared in the same manner as the post-incubation mixtures. The experiment included a positive control consisting of cell samples incubated with 25 μM hydrogen peroxide for 15 min on ice.

The comet assay was performed under alkaline conditions according to the procedure described by Tokarz et al. [77]. A freshly prepared cell suspension in 0.75% low melting point (LMP) agarose dissolved in PBS buffer was layered onto microscope slides pre-coated with 0.5% normal melting point (NMP) agarose. Cells were lysed for 1 h at 4 °C in a buffer containing 2.5 M NaCl, 0.1 M EDTA (ethylenediaminetetraacetic acid), 10 mM Tris, and 1% Triton X-100 (pH 10). After lysis, the slides were placed in an electrophoresis unit. DNA was allowed to unwind for 20 min in a solution containing 300 mM NaOH and 1 mM EDTA (pH > 13). Electrophoretic separation was performed in a solution containing 30 mM NaOH and 1 mM EDTA (pH >13) at 4 °C (the running buffer temperature did not exceed 12 °C) for 20 min at an electric field strength of 0.73 V/cm (28 mA). Subsequently, the slides were washed with water, drained, stained with 2 µg/mL DAPI (4′,6-diamidino-2-phenylindole dihydrochloride), and covered with coverslips. To prevent additional DNA damage, all procedures were conducted under limited light or in the dark.

Comets were observed at 200× magnification using an Eclipse fluorescence microscope (Nikon, Tokyo, Japan) equipped with a COHU 4910 video camera (Cohu Inc., San Diego, CA, USA) fitted with a UV-1 A filter block and connected to the personal computer-based image analysis system Lucia-Comet v. 7.0 (Laboratory Imaging, Prague, Czech Republic). One hundred images (comets) were randomly selected from each sample, and the mean percentage of DNA in the comet tail was taken as an index of DNA damage. The fluorescence intensity, which was proportional to the amount of DNA, was measured for the entire comet (head + tail) and separately for the tail. The percentage of DNA damage was calculated by dividing the tail fluorescence intensity by the total comet fluorescence intensity and multiplying the result by 100 [69,78].

DNA damage after incubation with the test materials is presented as the mean ± SEM. Statistical analysis was performed using a two-tailed Student’s *t*-test. Statistical significance levels refer to comparisons between the experimental samples (PLA and PLA-ALG-ZnS-HT) and the corresponding controls after the appropriate incubation time. Additionally, the positive controls were compared to their respective controls after 24 h of incubation. Each cell type had its own dedicated control group. Each cell type had its own dedicated control group. Differences were considered statistically significant at *p* < 0.001.

#### 3.2.11. Plasmid Relaxation Assay

To investigate the effects of the PLA-ALG-ZnS-HT composite on plasmid DNA, 1 cm^2^ pieces of each material were placed in separate wells of a 6-well plate and incubated with 3 mL of ultrapure water for 24 h at 37 °C in a 5% CO_2_. Following incubation, the resulting solutions were filtered through a 0.2 µm filter to ensure sterility. These filtered extracts were then combined with plasmid DNA at a 1:1 ratio to evaluate their impact on plasmid conformation.

The pUC19 plasmid was isolated from DH5α *Escherichia coli* using the AxyPrep Plasmid Miniprep Kit (Axygen Inc., Union City, CA, USA) according to the manufacturer’s instructions. Concentration and purity were assessed by measuring the A260/A280 ratio and agarose gel electrophoresis. Native pUC19 predominantly exists in a supercoiled form, which exhibits high electrophoretic mobility. To generate a linear form, the plasmid was digested with the restriction enzyme *Pst*I (New England Biolabs, Ipswich, MA, USA). The different topological forms (CCC and L) display distinct electrophoretic mobilities. Plasmid DNA (50 ng/µL) was incubated with the PLA and PLA-ALG-ZnS-HT composite post-incubation mixtures for 24 h. Samples were then subjected to 2% agarose gel electrophoresis, stained with ethidium bromide, and visualised under UV light (302 nm). Images were captured using a CCD (charge-coupled device) camera and analysed with GeneTools v.4.3.9.0. software (Syngene International Ltd., Baltimore, MD, USA). A 1 kb DNA ladder (GeneRuler 1 kb DNA Ladder, Thermo Fisher Scientific Inc., Waltham, MA, USA) was included to determine DNA fragment sizes.

## 4. Conclusions

This study demonstrated that it is possible to obtain alginate-functionalised PLA nonwovens incorporating zinc sulphide and hardystonite. This was achieved by melt-blowing PLA and subsequent dip-coating it in a sodium alginate solution, followed by treatment with zinc sulphide and hardystonite suspensions. Microscopic studies showed that the manufactured PLA-ALG-ZnS-HT composite was characterised by a typical three-dimensional fibrous structure, with randomly oriented nano- and microfibers creating a porous structure with interconnected pores. Zinc sulphide and hardystonite particles were successfully incorporated into the sodium alginate matrix, which formed a coating encapsulating the PLA fibres. The developed composite, containing a high amount of Zn, was characterised by higher specific surface area, total pore volume, and less negative zeta potential compared to the unmodified PLA nonwoven. Moreover, the high zinc content led to prolonged aPTT, likely due to the binding of XI, XII, and HK contact factors present in blood plasma. This implies that the PLA-ALG-ZnS-HT composite may function as an anticoagulant material. Furthermore, the PLA-ALG-ZnS-HT post-incubation mixtures did not exhibit cytotoxic or genotoxic effects against PBM and Hs68 cells. Future research should include more complex biological analyses to fully characterise the biological effects of these materials. In conclusion, the demonstrated safety of these materials supports their further evaluation in in vivo studies.

## Figures and Tables

**Table 1:** Specific surface area, total pore volume, and average pore size of the unmodified and modified PLA nonwovens.

Sample Name	Specific Surface Area [m^2^/g]	Total Pore Volume [cm^3^/g]	Average Pore Size [nm]
PLA	1.082	3.985 × 10^−3^	14.73
PLA-ALG-ZnS-HT	1.217	8.210 × 10^−3^	26.99

**Table 2:** Parameters used for PLA nonwoven fabrication via the melt-blown process.

Values	Processing Parameters
195 °C	Extruder temperature—zone 1
245 °C	Extruder temperature—zone 2
260 °C	Extruder temperature—zone 3
260 °C	Die head temperature
260 °C	Air heater temperature
7–8 m^3^/h	Airflow rate
95 g/m^2^	Basis weight of nonwovens
6 g/min	Polymer throughput

## Data Availability

The data are included in the text.
